# Estimating loss in capability wellbeing in the first year of the COVID-19 pandemic: a cross-sectional study of the general adult population in the UK, Australia and the Netherlands

**DOI:** 10.1007/s10198-022-01498-y

**Published:** 2022-07-24

**Authors:** Paul Mark Mitchell, Rachael L. Morton, Mickaël Hiligsmann, Samantha Husbands, Joanna Coast

**Affiliations:** 1 Health Economics Bristol (HEB), Population Health Sciences, Bristol Medical School, University of Bristol, 1-5 Whiteladies Road, Bristol, BS8 1NU UK; 2 NHMRC Clinical Trials Centre, Faculty of Medicine and Health, University of Sydney, Camperdown, Australia; 3 Department of Health Services Research, CAPHRI Care and Public Health Research Institute, Maastricht University, Maastricht, the Netherlands

**Keywords:** Health economics, COVID-19, Wellbeing, Capability approach

## Abstract

**Objectives:**

To estimate capability wellbeing lost from the general adult populations in the UK, Australia and the Netherlands in the first year of the COVID-19 pandemic and the associated social restrictions, including lockdowns.

**Design:**

Cross-sectional with recalled timepoints.

**Setting:**

Online panels in the UK, Australia and the Netherlands conducted in February 2021 (data collected 26 January–2 March 2021).

**Participants:**

Representative general adult (≥ 18 years old) population samples in the UK (*n* = 1,017), Australia (*n* = 1,011) and the Netherlands (*n* = 1,017)

**Main outcome measure:**

Participants completed the ICECAP-A capability wellbeing measure in February 2021, and for two recalled timepoints during the initial lockdowns in April 2020 and in February 2020 (prior to COVID-19 restrictions in all three countries). ICECAP-A scores on a 0–1 no capability–full capability scale were calculated for each timepoint. Societal willingness to pay estimates for a year of full capability (YFC) was used to place a monetary value associated with change in capability per person and per country. Paired *t* tests were used to compare changes in ICECAP-A and YFC from pre- to post-COVID-19-related restrictions in each country.

**Results:**

Mean (standard deviation) loss of capability wellbeing during the initial lockdown was 0.100 (0.17) in the UK, 0.074 (0.17) in Australia and 0.049 (0.12) in the Netherlands. In February 2021, losses compared to pre-lockdown were 0.043 (0.14) in the UK, 0.022 (0.13) in Australia and 0.006 (0.11) in the Netherlands. In monetary terms, these losses were equivalent to £14.8 billion, AUD$8.6 billion and €2.1 billion lost per month in April 2020 and £6.4 billion, A$2.6 billion and €260 million per month in February 2021 for the UK, Australia and the Netherlands, respectively.

**Conclusions:**

There were substantial losses in capability wellbeing in the first year of the COVID-19 pandemic. Future research is required to understand the specific impact of particular COVID-19 restrictions on people’s capabilities.

**Supplementary Information:**

The online version contains supplementary material available at 10.1007/s10198-022-01498-y.

## Introduction

The COVID-19 pandemic has, internationally, led to a variety of restrictions on daily living across the general population in a bid to mitigate its spread [1]. Although there is a general acknowledgement that the impact of COVID-19 is not only in terms of the physical health impact of the disease, it has been suggested that public health policy has largely been informed by a narrow epidemiological view of minimising COVID-19 cases and ultimately deaths due to COVID-19 [2]. Quality of life impacts of restrictions imposed on daily living across the general population to tackle COVID-19 have received less attention from many health scientists with priority given to averting deaths from COVID-19 and latterly to concerns related to long COVID [3].

The COVID-19 pandemic has also highlighted the difficulty of relying on traditional economic indicators and has led to renewed calls for wellbeing to be at the heart of policy-making [4, 5]. Such ideas were originally proposed by government leaders, such as by the UK and French governments in the early 2010s, but their implementation in practice has remained limited [6]. A wellbeing budget has recently been implemented by the New Zealand government, leading to greater priority in the provision of mental health services as a result of the estimated impact on improving wellbeing [7]. Other governments are also beginning to adopt a similar wellbeing economy approach to decide how best to allocate their budgets, including in Finland, Iceland, Scotland and Wales [8]. In July 2021, the UK government added supplemental guidance to their green book guide for economic appraisals of policies, programmes and projects, allowing for the inclusion of wellbeing in cost–benefit analysis [9]. Wellbeing indicators offer the advantage of cross-sector comparisons that are currently incompatible with most evaluations undertaken in the health sector, where these rely on the health focused quality adjusted life year (QALY) metric for cost-effectiveness analysis [10]. Indicators like the QALY or the Value of a Statistical Life Year are commonly used to quantify the monetary value associated with policy impacts on morbidity and mortality [11].

There were notable divergences in national policies in tackling COVID-19 through non-pharmaceutical interventions during the first year of the pandemic [12] and prior to the availability of effective COVID-19 vaccines. These approaches have generally been grouped in terms of elimination and mitigation strategies [13], albeit with much variation in the implementation of such strategies across different countries. Given the large impact across the general population from such policies, it is important to assess the wellbeing impact of such interventions and strategies on their respective populations.

One way of assessing population impacts in wellbeing terms has been developed to capture people’s capabilities to do and be things in life that matter to them [14], inspired by the works of economist and philosopher Amartya Sen [15–17]. The ICECAP-A is a short validated five attribute measure that captures capabilities, previously identified as being important to people’s wellbeing [18]. It has been used in a variety of general adult and patient populations internationally [19], with capability wellbeing measures now recommended for use in economic assessments of social care interventions in England [20] and for long-term care in the Netherlands [21]. Given the restrictions on people’s capabilities during the COVID-19 pandemic, it provides an appropriate lens through which to assess the quality of life impacts on the general population.

This study aims to quantify the impacts of COVID-19 restrictions on general adult population capability wellbeing in the UK, Australia and the Netherlands. There was a notable divergence in the policies pursued in the three countries during the first year of the pandemic. The Australian government pursued a COVID-19 elimination strategy through regional lockdowns and strict international quarantine rules, whereas the UK and the Netherlands pursued policies on mitigating the spread of COVID-19, which also included variations of lockdowns and social restrictions when COVID-19 case rates deemed such action to be required [1].

## Methods

### Contextual background and Study design

We undertook a cross-sectional study in February 2021 (data collected between 26 January and 2 March 2021) in the UK, Australia and the Netherlands. The ICECAP-A measure has been validated in these three high-income countries [22–24], and this was the primary rationale for choosing these countries. During this time period, there were notable differences in COVID-19-related measures in place. The UK was in a third national lockdown following the emergence of the alpha variant in the UK in December 2020. The Netherlands was also in a lockdown since December 2020, but primary schools and care centres had just reopened in that month. Australia had reduced many domestic restrictions by February 2021, albeit international travel was largely limited, with two-week hotel quarantine rules for arrivals not from Australia or New Zealand and regional lockdowns enacted when any COVID-19 cases emerged.

We used online panel surveys (PureProfile for UK and Australia, Panel Inzicht for the Netherlands) to recruit a sample of 1,000 participants in each country. This is a sample size commonly used when undertaking nationally representative polling surveys, where further sampling is unlikely to increase the precision of summary estimates [25]. National samples were quota sampled to be representative in terms of age, sex, minority ethnicity, education level and national regions. Participants were asked to complete questions related to their capability wellbeing, socio-demographic variables and other COVID-19 relevant questions. Surveys were completed through panel survey online platforms, using a desktop, laptop or tablet computer, to minimise any potential influence a different mode of administration (i.e. smart phones) may have on responses. Participants were excluded if they did not meet one of the quota requirements (e.g. under 18 years old), a quota requirement was full (e.g. age group) or they completed the survey in too short a time period (i.e. 30% of the median time to complete the survey based on a soft launch of 100 participants per country). As is typical with online panel surveys, completing the survey implies consent for the data collected to be used for research purposes. Ethical approval for this study was granted by the University of Bristol Faculty of Health Science Research Ethics Committee (reference number: 111902).

### Variables

#### ICECAP-A

The ICECAP-A is a short, self-complete five attribute measure that assesses individual capability in terms of stability (feeling settled and secure), attachment (love, friendship and support), autonomy (independence), achievement (achieve and progress) and enjoyment (enjoyment and pleasure). These five attributes were identified through qualitative research with members of the UK general adult population to establish what was most important to them in their quality of life [18]. Each attribute consists of four levels of capability, ranging from no (1) to full (4) capability. The ICECAP-A has been validated for use in general adult population settings [22], as well as in specific patient populations and countries, including Australia [19]. A validated Dutch translation of the ICECAP-A [24, 26] was used in the Dutch sample.

The three ICECAP-A versions were completed by all participants in reverse chronological order in an attempt to minimise recall bias influencing the reporting of current levels of capability wellbeing. The three versions completed were as follows:

1. The ICECAP-A original version, where individuals are asked to “describe your quality of life **AT THE MOMENT**”
2. First lockdown—“describe your quality of life **DURING THE INITIAL CORONAVIRUS RESTRICTIONS IN APRIL 2020**”
3. Pre-lockdown—“describe your quality of life **BEFORE THE CORONAVIRUS RESTRICTIONS IN FEBRUARY 2020**”

#### COVID-19 stringency index

Separate to this study, the Oxford Coronavirus Government Response Tracker project calculated a Stringency Index, a composite measure of nine response metrics to measure the strictness of government policies. The nine metrics are: school closures, workplace closures, cancellation of public events, restrictions on public gatherings, closures of public transport, stay-at-home requirements, public information campaigns, restrictions on internal movements and international travel records. The index on any given day is calculated as the mean score of the nine metrics on a 0–100 scale with 100 equalling the strictest response [1]. We calculate a monthly mean stringency index score based on the daily stringency index score in each country in April 2020 and February 2021 to descriptively compare the extent of restrictions with general population capability wellbeing.

### Statistical analysis

A 0–1 no capability–full capability ICECAP-A summary score can be generated based on the relative importance of each attribute and each level of capability within the attribute according to average estimates of their importance according to the general population in the UK [27] and the Netherlands [28]. No Australian estimate is currently available, so we applied the UK general population values to the Australian sample. A sensitivity analysis using the UK value set in the Dutch sample was also conducted. To explore the areas of capability wellbeing that have been most affected by the COVID-19 pandemic and the associated restrictions, means for each individual four-level attribute on the ICECAP-A (ranging from 1–4 no capability–full capability) at each time point are also given.

ICECAP-A summary scores can be combined over time to give an outcome of years of full capability (YFC) equivalent, ranging from 0–1 per year where 0 represents no capability for a year and 1 represents full capability for a year [29]. For each completion of the ICECAP-A, we assume capability levels were constant for the month in question. We compare change in YFC per month from February 2020 (*pre-lockdown*) to April 2020 (*first lockdown*), and from February 2020 to February 2021 (*restrictions one year in*), as follows in Eq. 1:

1 $${\text{YFC}}\;{\text{change}}\;{\text{per}}\;{\text{month}}\;{\text{in}}\;{\text{country}}_{i,j,k} = ((\overline{x}\;{\text{ICECAP-A}}\;{\text{score}}\;\left( {t_0 } \right) \pm \overline{x}\;{\text{ICECAP-A}}\;{\text{score}}\;\left( {t_{{1},{2}} } \right)) \div {12}) \times {\text{country}}_{i,j,k} \;{\text{adult}}\;{\text{population}}$$

where *x̅* is the sample mean for given *t*; *t*_0_ is the February 2020 (pre-lockdowns), *t*_1_ is the April 2020 (first lockdowns), *t*_2_ is the February 2021 (restrictions 1 year in).

To do this, we extrapolate our sample levels to the adult population in each country based on national statistical office estimates for 2020 [30–32], respectively. From Eq. 1, we are then able to estimate a monetary value associated with the change in YFC per month as follows in Eq. 2:

2 $${\text{Monetary}}\;{\text{value}}\;{\text{of}}\;{\text{YFC}}\;{\text{lost}}\;{\text{per}}\;{\text{month}}\;{\text{in}}\;{\text{country}}_{i,j,k} = {\text{Eq}}\,1. \times {\text{WTP}}\;{\text{for}}\;{\text{YFC}}$$

where WTP is the willingness to pay for a YFC in country _i,j,k_

Societal willingness to pay for a YFC has recently been estimated at £33,500–£36,150 in the UK [33]. This study uses the lower figure (i.e. £33,500) to provide the most conservative estimate. We apply the 2020 Organisation for Economic Co-operation and Development (OECD) purchasing price parity estimates [34] to adjust the UK willingness to pay figure to generate comparable estimates in local currencies. We also produce monetary estimates for the average adult in each country in UK sterling (£), to aid comparison across countries.

Paired t-tests were used to estimate the change in each of ICECAP-A attribute levels, ICECAP-A scores and YFC per month between the *pre-lockdown* estimate in February 2020 and: (1) *first lockdowns* in April 2020 and (2) *restriction one year in* in February 2021. 95% confidence intervals are reported for changes over time for all mean estimates. Analysis was undertaken using Stata version 16.

## Results

In total, 1,799 participants in the UK, 2,426 in Australia and 3,646 in the Netherlands attempted the survey. The notably higher number of participants approached in the Netherlands was due to the Dutch panel being underrepresented in terms of minority ethnicity status, resulting in this quota being lifted, and the education question (i.e. age when finished full-time education) required further refinement to appropriately stratify the Dutch sample based on their secondary-level education system often not finishing before students were 18 years old. The main reasons for not being included in the final sample were being over the quota (UK *n* = 679, Australia *n* = 1,298 and the Netherlands *n* = 1,201), followed by survey incomplete (UK *n* = 100, Australia *n* = 106, the Netherlands *n* = 1,120) and screened out (UK *n* = 3, Australia *n* = 11, the Netherlands *n* = 308).

Table 1 describes the characteristics of the sample. In total (percentage of participants who attempted to complete the survey), 1,017 (57%) participants in the UK, 1,011 (42%) participants in Australia and 1,017 (28%) participants in the Netherlands were included in the analysis. Quota samples for age, sex, major geographical region, minority ethnicity status and education level were reached in all countries except for minority ethnicity status in the Netherlands, which was underrepresented.

**Table 1:** Quota target characteristics of sample

	UK	Quota	Australia	Quota	Netherlands	Quota
Total	1017	1000	1011	1000	1017	1000
Female	517	506	513	508	499	503
Male	499	494	498	492	516	497
Other	1		0		2	
Minority ethnicity	123	130	217	240	77	230
Majority ethnicity	873	870	767	760	922	770
Prefer not to say	21		27		18	
Higher education	439	420	433	420	389	340
Lower education	578	580	578	580	628	660
Age groups						
18–24	113	108	116	116	109	109
25–34	163	172	200	191	146	160
35–44	162	160	174	172	156	147
45–54	181	175	167	162	169	171
55–64	160	152	142	149	178	169
65–74	134	127	120	118	150	139
75+	104	106	92	92	109	105
Regions/states/territories						
East Midlands	75	73				
East of England	98	93				
London	129	134				
North East	40	40				
North West	108	110				
South East	145	137				
South West	84	84				
West Midlands	86	89				
Yorkshire & the Humber	86	82				
Northern Ireland	31	28				
Scotland	85	82				
Wales	50	48				
Australian Capital Territory			18	17		
New South Wales			324	318		
Northern Territory			10	10		
Queensland			196	201		
South Australia			74	69		
Tasmania			21	21		
Victoria			261	260		
Western Australia			107	104		
Noord Nederland					111	100
Oost Nederland					213	211
West Nederland					475	478
Zuid Nederland					218	211

Quotas reported above are the point estimates (within acceptable ranges that were provided to the panel survey company). These estimates were taken from each national statistics agency, as well as the OECD for higher education attainment statistics

All countries recorded a similar average monthly stringency index score during the first lockdown, with the UK scoring 80, the Netherlands 79 and Australia 72. By February 2021, the UK recorded a stringency index score of 88, the Netherlands at 80 and Australia at 63 [1].

**Fig. 1:**
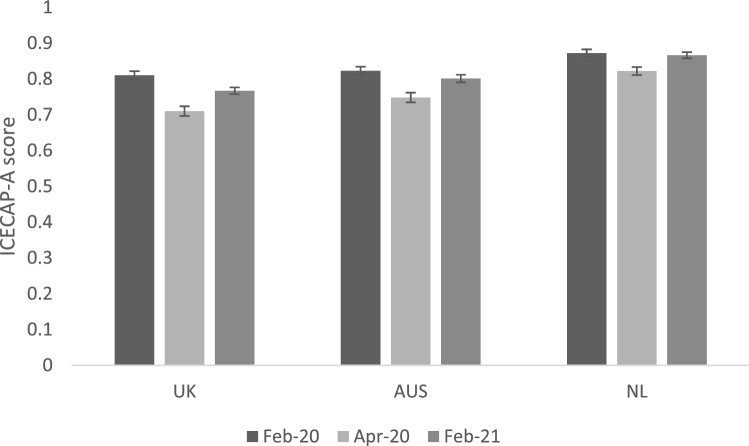
Average ICECAP-A scores pre-lockdown, first lockdowns and one year into COVID-19 restrictions. ICECAP-A scores range from 0–1 no capability-full capability. Error bars indicate standard deviations for means. *Feb* February, *Apr* April, *UK* United Kingdom (*n* = 1,017), *AUS* Australia (*n* = 1,011), *NL* the Netherlands (*n* = 1,017). Bars represent 95% confidence intervals around the mean estimate

Figure 1 reports the mean ICECAP-A scores for February 2021 (one year into COVID-19 restrictions) and the two recalled time points of February 2020 (pre-lockdown) and April 2020 (initial lockdown). Pre-lockdowns, the Netherlands had the highest mean (standard deviation) capability wellbeing of 0.871 (0.15), followed by Australia at 0.823 (0.18), with the UK average capability wellbeing lowest at 0.810 (0.19). Mean UK capability wellbeing fell furthest during the initial lockdowns to 0.710 (0.22), with Australia at 0.748 (0.22) and the Netherlands at 0.822 (0.17). All countries saw improvements in mean capability wellbeing in February 2021 compared to April 2020, but all remained lower than the capability estimates for February 2020. In February 2021, where the Netherlands again reported the highest average capability levels at 0.865 (0.14), Australia was next at 0.801 (0.19), and the UK was lowest with 0.767 (0.19).

Comparing the mean ICECAP-A scores in countries across the three timepoints resulted in an average loss of capability wellbeing between pre- and post-COVID-19 restrictions at all timepoints in all three countries. During the initial lockdown, mean (standard deviation) capability wellbeing loss was 0.100 (0.17) in the UK, 0.074 (0.17) in Australia and 0.049 (0.12) in the Netherlands. In February 2021, losses compared to pre-lockdown were 0.043 (0.14) in the UK, 0.022 (0.13) in Australia and 0.006 (0.11) in the Netherlands.

**Fig. 2:**
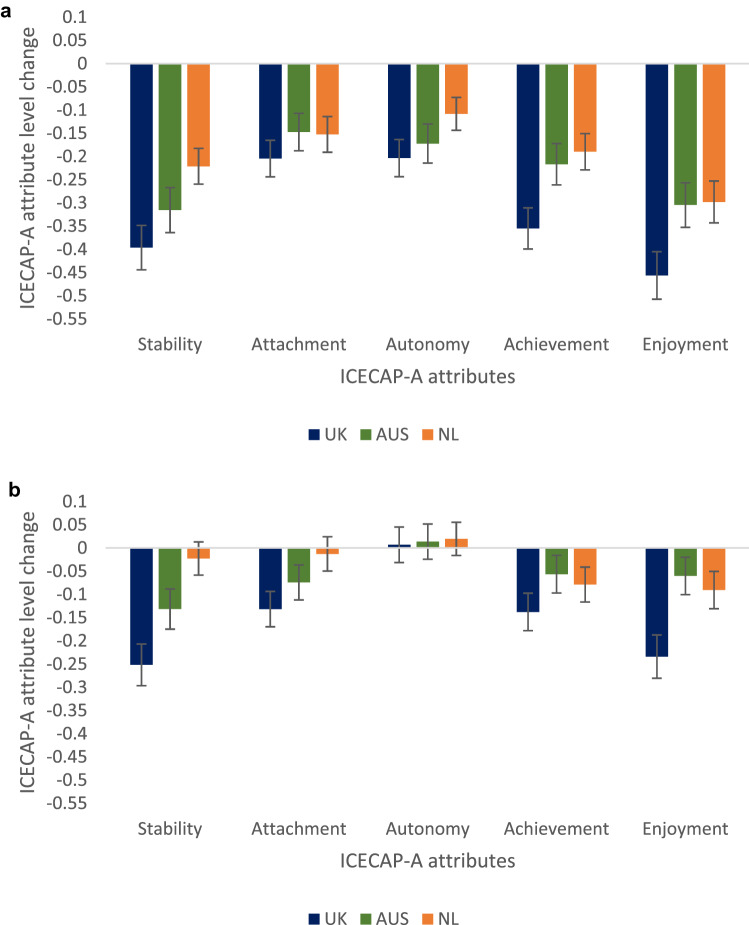
**a** Mean change in ICECAP-A attribute levels from pre-lockdown to initial lockdown. ICECAP-A attribute levels range from 1 (no capability) to 4 (full capability). *UK* United Kingdom (*n* = 1,017), *AUS* Australia (*n* = 1,011), *NL* the Netherlands (*n* = 1,017). Bars represent 95% confidence intervals around the mean estimate. **b** Mean change in ICECAP-A attribute levels from pre-lockdown to restrictions 1 year on. ICECAP-A attribute levels range from 1 (no capability) to 4 (full capability). *UK* United Kingdom (*n* = 1,017), *AUS* Australia (*n* = 1,011), *NL* the Netherlands (*n* = 1,017). Bars represent 95% confidence intervals around the mean estimate

Figure 2a shows the change in ICECAP-A attributes from February 2020 to April 2020. All attributes saw a statistically significant fall in mean capabilities in all countries in April 2020. The largest falls in capability were seen in the *stability* and *enjoyment* attributes in all three countries. Figure 2b shows the change in ICECAP-A attributes from February 2020 to February 2021. Levels of *autonomy* recovered to February 2020 levels in all three countries by February 2021, but all other attributes remained statistically significantly lower than their February 2020 levels in both Australia and the UK. *Achievement* and *enjoyment* were the only attributes that were statistically significantly lower in the Netherlands by this time.

Table 2 reports on the estimated loss of capability wellbeing and monetary value associated with differences of YFC per month when comparing February 2020 to April 2020 and February 2020 to February 2021 in each country. Monetary estimates associated with losses in YFC per month from pre-lockdown in February 2020 to April 2020, during the initial lockdowns, amount to £14.8 billion in the UK, AUD$8.6 billion in Australia and €2.1 billion in the Netherlands. In February 2021, one year into COVID-19 restrictions, loss in YFC per month was £6.4 billion in the UK, AUD$2.6billion in Australia and €260 million in the Netherlands when compared to pre-lockdown estimates in February 2020.

**Table 2:** Loss in capability wellbeing, years of full capability and monetary value estimates compared to pre-lockdowns in February 2020

	UK			Australia			the Netherlands		
	Central estimate	95% lower C.I	95% higher C.I	Central estimate	95% lower C.I	95% higher C.I	Central estimate	95% lower C.I	95% higher C.I
Adult Population^a^	52,890,004			19,753,735			14,069,000		
ICECAP-A score^b^ mean reduction since Feb 2020									
Apr-20	0.100	(0.090	0.111)	0.074	(0.064	0.084)	0.049	(0.042	0.057)
Feb-21	0.043	(0.035	0.052)	0.022	(0.014	0.030)	0.006	(-0.001	0.013)
YFC^c^ lost per month per country									
Initial lockdown	440,750	(396,675	489,233)	121,815	(105,353	138,276)	57,448	(49,242	66,828)
Restrictions 1 year in	189,523	(154,263	229,190)	36,215	(23,046	49,384)	7,035	(-1,172	15,241)
Monetary value of YFC lost per month (billions)^d^									
Apr-20	£14.77	(£13.29	£16.39)	A$8.59	(A$7.43	A$9.75)	€ 2.13	(€ 1.82	€ 2.47)
Feb-21	£6.35	(£5.17	£7.68)	A$2.56	(A$1.62	A$3.48)	€ 0.26	(-€ 0.04	€ 0.56)

^a^Adult population estimates are for 2020 from the relevant national statistics authorities in the respective countries [30–32]

^b^ICECAP-A score on a 0–1 no capability-full capability scale [27]

^c^YFC, Years of full capability = 1 when ICECAP-A score is 1 for a full year and 0 when ICECAP-A score is 0 for a full year [29]

^d^UK societal willingness to pay for a YFC[33] (£33,500) and OECD 2020 purchasing power parity adjusted estimates for Australia (AUD$70,475) and the Netherlands (€37,025) [34]

**Fig. 3:**
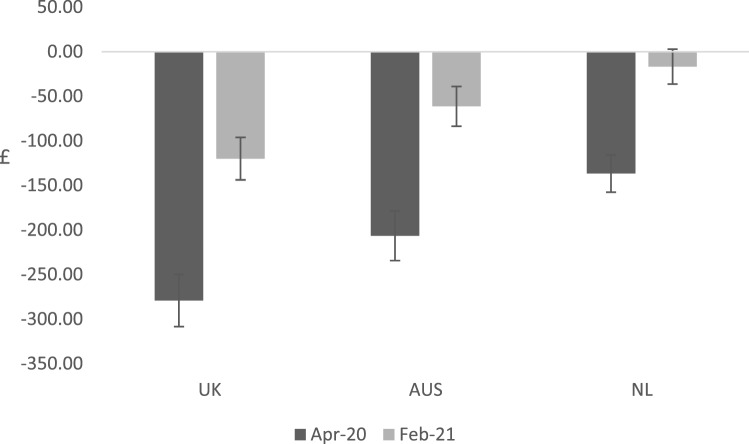
Value (£) per average adult associated with loss of capability wellbeing per month during the first lockdowns and one year into restrictions. Bars represent 95% confidence intervals around the mean estimate. *UK* United Kingdom, *AUS* Australia, *NL* the Netherlands

In Fig. 3, the mean (standard deviation) monetary loss per person in each country during the first lockdowns and one year into the restrictions is illustrated. During the first lockdowns, the monetary loss per person associated with a reduction in YFC per month was £280 (484) in the UK, £207 (462) in Australia and £137 (346) in the Netherlands. One year into restrictions, falls in YFC per month were valued at £121 (388) in the UK for each individual, £60 (360) in Australia and £17 (298) in the Netherlands.

The sensitivity analysis using UK general population values in the estimation of the ICECAP-A scores for the Dutch sample compared similarly for all analysis (see Online Resource 1).

## Discussion

### Statement of principal findings

This study has explored the impact on capability wellbeing in the first year of the COVID-19 pandemic in the general adult populations in the UK, Australia and the Netherlands. It highlights large losses in capability wellbeing for the general adult population that occurred in the initial lockdowns in all countries and similarly large losses in the UK during its third lockdown in February 2021 to tackle the spread of the alpha variant. In monetary terms, we estimate the largest losses in YFC to be £14.8 billion per month in the UK during the initial lockdown, more than the estimated monthly spend on health by the UK government in the 2019/20 financial year [35]. Even in Australia in February 2021, where fewer domestic restrictions were in place compared to European countries [1], average levels of adult capability wellbeing were still down on estimated levels before COVID-19 and the associated restrictions were put in place. The Netherlands, which, like the UK, was in a form of lockdown during February 2021, managed to get closest to restoring average adult capability scores to pre-lockdown levels.

### Strengths and weaknesses of the study

To our knowledge, this study represents the first estimate of the losses of wellbeing in terms of people’s capability to do and be things in life that matter to them at different stages during the first year of the COVID-19 pandemic. It provides information for three high income countries. The samples were nationally representative in terms of age, sex, education level and geographical spread across each of the three countries. Minority ethnicity status was also represented in the UK and Australian sample, but the Dutch sample was underrepresented in this area. As well as this, and like all panel surveys, the population can be representative of certain characteristics, but may miss groups who are less likely to join internet panels in the first place, such as those who do not or cannot access the internet easily from their place of residence.

This study also relies on retrospective recall in February 2021 for capability wellbeing levels in February 2020 and April 2020. We are aware that this is a sub-optimal approach when compared to prospective data collection, although both approaches are open to different forms of bias [36]. Nonetheless, retrospective recall can be particularly useful in situations where prospective data collection is not available [37]. Although the use of retrospective recall was not ideal, it was unavoidable given the circumstances, and reassurance about the reliability of the data can be obtained in considering previous studies. First, comparable estimates of general population capability wellbeing levels have been found previously in the UK to those recalled retrospectively for February 2020 here [22, 38]. Second, a higher average capability wellbeing level has been observed in Australia compared to the UK previously [39]. One concern with retrospective recall is an overestimation of past quality of life levels [40]. However, given that pre-lockdown average levels in this study are comparable to pre-lockdown population norms, and initial lockdown estimates in April 2020 are lower than our prospective estimates for February 2021 in all countries, this suggests that the retrospective recall has not distorted the findings reported here. The valuation estimates for the ICECAP-A scores and the willingness to pay for a YFC are based on UK sample estimates for Australia, as these were the only estimates available when the analysis was undertaken, so caution is required when interpreting Australian estimates.

A key strength of our study is the ability to quantify a capability wellbeing estimate at a general adult population level using a validated five attribute capability measure, the ICECAP-A [18, 19]. This can then be used to generate an outcome metric over time, YFC, that can inform policies on their relative merits in monetary terms. Such an outcome is not dissimilar to estimates typically used in health economics to inform cost-effectiveness of health interventions, such as QALYs [41], but has a much broader wellbeing basis for estimation. One advantage of using YFC is that it allows for comparisons across health and other parts of the economy that are currently difficult to compare when using the health centric QALYs [10]. We are, however, only able to describe what the capability wellbeing levels were at different time points during the first year of the COVID-19 pandemic in this study and cannot directly assess specific policies that were introduced during this time period. Collecting such data in nationally representative panel surveys on a regular basis would have provided us with more detailed estimates of changes in capability wellbeing during the first year of the COVID-19 pandemic. Such data would have allowed us to generate national YFC estimates for a full year and not just monthly estimates and comparisons we are limited to with the data we have here. We also only focus on capability wellbeing of adults, yet capability wellbeing in children and young people has also been impacted by COVID-19 and related government policies. Our estimates therefore are almost certainly conservative estimates of the capability wellbeing lost across the total population in these three countries.

### Meaning of the study: possible explanations and implications for policymakers

The main finding from this study is the detrimental impact that the combined effect of COVID-19, its associated restrictions (in particular the first lockdowns), and the elimination and mitigation policies put in place by these Governments have had on general adult population capability wellbeing levels. The 0.1 fall on the ICECAP-A index in the UK from February 2020–April 2020 (see Table 2) is comparable to the difference in ICECAP-A observed elsewhere in healthy people to those with a primary health condition of arthritis, asthma, cancer, diabetes or coronary heart disease [42]. In monetary terms, these losses quantified in terms of YFC loss are substantial, and even in Australia in February 2021 where there was more success in minimising the spread of COVID-19 and fewer domestic restrictions had been implemented compared to the two European countries during this time [1], the country was still estimated to be losing YFC at a rate of AUD$2.6 billion per month when compared to pre-COVID-19 levels.

There is also a notable difference in average capability wellbeing losses per person across the timepoints in the study. What this suggests is that all three governments did better over time at reducing the capability wellbeing losses, yet some countries appeared to do better than others at getting closer to pre-lockdown levels of capability. This suggests that some of the policies pursued in each country were likely to have bigger positive (e.g. the opening of schools and childcare provision in the Netherlands in February 2021) and negative (e.g. the strict international travel rules in Australia) impacts on general adult population levels of capability wellbeing. Although it is not possible to completely disentangle policies pursued from the differing impact of COVID-19 across places and populations in this study, these findings may be particularly pertinent for policymakers to take account of as they develop policy to deal with new variants of COVID-19 that have the potential for vaccine escape.

### Unanswered questions and future research

It is important to state, however, that this study does not provide evidence of the relative success or failure of different national government policies in tackling COVID-19. Indeed, even though the initial lockdowns were particularly costly in terms of YFC lost, without those measures more people are likely to have lost their lives due to COVID-19 and associated YFC. Some attempts have been made to reconcile economic and epidemiological modelling in assessing different COVID-19 policies [43], but none have attempted to quantify outcomes in terms of YFC [44], and this may prove a useful area for future research. Further research is also required to explore the variation in capability wellbeing impacts across different members of society. A comparison of capability wellbeing with measures of health status and subjective wellbeing during the COVID-19 pandemic may also be useful to help decision-makers choose appropriate tools to aid their decision-making on the quality of life impacts across the general population during pandemics and social restrictions. We would also recommend future general population analysis to account for the impact on children and young people’s capabilities, with measures in this area currently under development [45, 46].

## Conclusion

COVID-19 and government-related policies have led to significant falls in general adult capability wellbeing levels in the UK, Australia and the Netherlands in the first year of the pandemic. Future government pandemic policy-making should consider the impact such decisions are having on their citizens’ ability to do and be things in life that matter to them, alongside the direct health impacts of diseases like COVID-19. We provide a method to quantify YFC and to convert that outcome into a meaningful monetary estimate that can be used in assessing the cost-effectiveness of public health pandemic-related policies that have impacts across different sectors of society.

## Supplementary Information

Below is the link to the electronic supplementary material.

Supplementary file1 (PDF 166 KB)

## Data Availability

Data are available on request at the University of Bristol data repository at 10.5523/bris.v6rsovuym4lv2iaeteqka76ur.
