# Supplementary material for: Estimating loss in capability wellbeing in the first year of the COVID-19 pandemic: a cross-sectional study of the general adult population in the UK, Australia and the Netherlands
Source: Eur J Health Econ. 2022 Jul 24;24(4):609–19. doi: 10.1007/s10198-022-01498-y (PMC9308953; doi:10.1007/s10198-022-01498-y)

Online Resource 1. Sensitivity Analysis Figures and Table

Estimating loss in capability wellbeing in the first year of the COVID-19 pandemic: a cross-sectional study of the general adult population in the UK, Australia, and the Netherlands

European Journal of Health Economics

Paul Mark Mitchell*, Rachael L. Morton, Mickaël Hiligsmann, Samantha Husbands, Joanna Coast

*Corresponding author: paul.mitchell@bristol.ac.uk

Health Economics Bristol (HEB), Population Health Sciences, Bristol Medical School, University of Bristol, 1-5 Whiteladies Road, Bristol, BS8 1NU, UK.

doi: https://doi.org/10.1007/s10198-022-01498-y

All analysis presented in this supplementary material is a replication of the same tables and figures from the main paper. The letter S is added to the front of the relevant Figure or Table so they can be compared with the original numbering in the main paper. The sensitivity analysis presented here uses the UK general population value set for all ICECAP-A summary score estimates. In the main paper, the Dutch value set was used in the Netherlands.

Figure S1. Average ICECAP-A scores pre-lockdown, first lockdowns and one year into COVID-19 restrictions

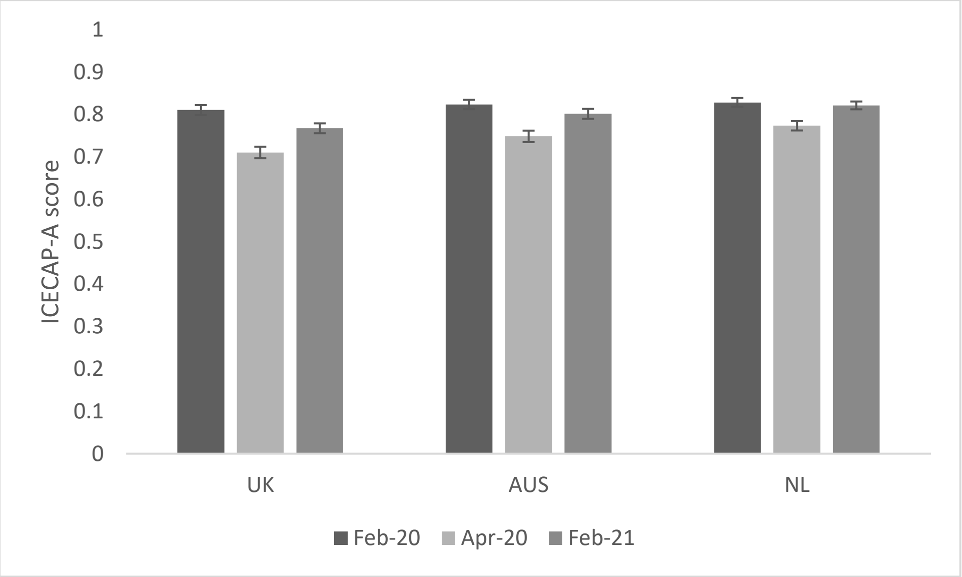

Table S2. Loss in capability wellbeing, years of full capability and monetary value estimates compared to pre-lockdowns in February 2020

	UK			Australia			the Netherlands		
	central estimate	95% lower C.I.	95% higher C.I	central estimate	95% lower C.I.	95% higher C.I	central estimate	95% lower C.I.	95% higher C.I
Adult Population[i]		52,890,004			19,753,735			14,069,000	
ICECAP-A score[ii] mean reduction since Feb 2020									
Apr-20	0.100	(0.090	0.111)	0.074	(0.064	0.084)	0.055	(0.047	0.063)
Feb-21	0.043	(0.035	0.052)	0.022	(0.014	0.030)	0.008	(-0.001	0.013)
YFC[iii] lost per month per country									
Initial lockdown	440,750	(396,675	489,233)	121,815	(105,353	138,276)	64,483	(55,104	73,862)
Restrictions 1 year in	189,523	(154,263	229,190)	36,215	(23,046	49,384)	9,379	(1,172	17,586)
Monetary value of YFC lost per month (billions)[iv]									
Apr-20	£14.77	(£13.29	£16.39)	A$8.59	(A$7.43	A$9.75)	€ 2.39	(€ 2.04	€ 2.73)
Feb-21	£6.35	(£5.17	£7.68)	A$2.56	(A$1.62	A$3.48)	€ 0.26	(€ 0.04	€ 0.65)

Figure S3. Value (£) per average adult associated with loss of capability wellbeing per month during the first lockdowns and one year into restrictions

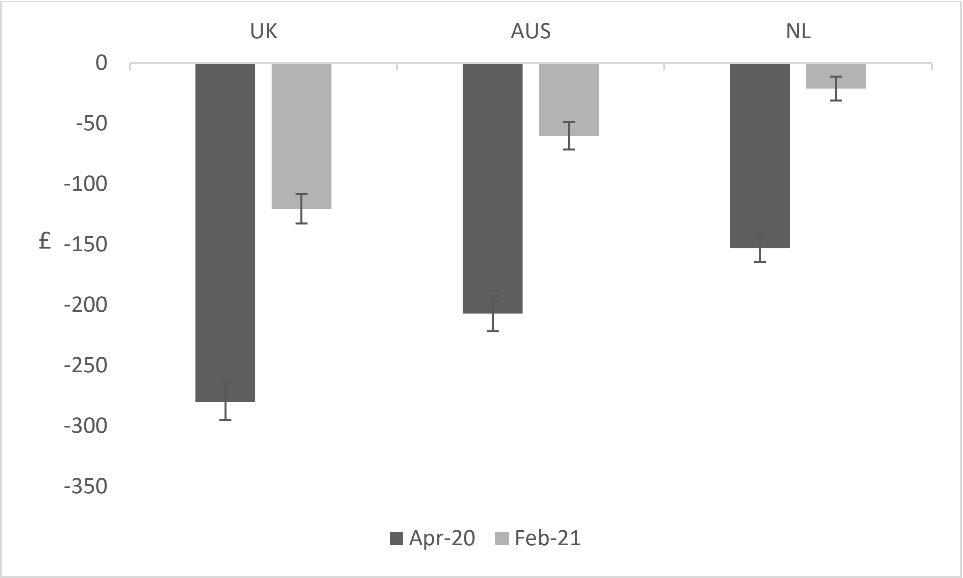

Supplement: Supplementary file 1 — Supplementary file1 (PDF 166 KB) [file 10198_2022_1498_MOESM1_ESM.pdf]
